# A double-blind, randomized controlled trial to explore oral tranexamic acid as adjunct for the treatment for postpartum hemorrhage

**DOI:** 10.1186/s12978-020-0887-2

**Published:** 2020-03-06

**Authors:** Ayisha Diop, Dina Abbas, Nguyen thi Nhu Ngoc, Roxanne Martin, Ange Razafi, Hoang Thi Diem Tuyet, Beverly Winikoff

**Affiliations:** 1 Gynuity Health Projects, 220 E. 42nd Street Suite 710, New York, NY 10017 USA; 2 Center for Research and Consultancy in Reproductive Health (CRCRH), 38A Nguyễn Lâm. Ward 6 District 10, Ho Chi Minh City, Vietnam; 3 Maternité Hopital Regional El Hadji Ahmadou Sakhir Ndieguene De Thiès, Thiès, Senegal; 4 Hung Vuong Hospital, Hong Bang, Ward 12, District 5, Ho Chi Minh City, Vietnam

**Keywords:** Postpartum hemorrhage (PPH), Tranexamic acid, Misoprostol

## Abstract

**Background:**

Oral tranexamic acid (TXA), if effective in reducing blood loss after delivery for women experiencing primary PPH, could be administered where parenteral administration is not feasible. This trial assessed the efficacy, safety, and acceptability of oral TXA when used as an adjunct to sublingual misoprostol to treat postpartum hemorrhage (PPH) following vaginal delivery.

**Methods:**

From October 2016 to January 2018, women presenting at four hospitals in Senegal and Vietnam for vaginal delivery were screened for enrollment in the trial. Women diagnosed with postpartum hemorrhage (defined as blood loss ≥700 ml) were randomized to receive either oral TXA (1950 mg) or placebo in addition to 800 mcg sublingual misoprostol. Postpartum blood loss was measured using a calibrated drape. Blood loss for all PPH cases was recorded for 2 h after administration of the drugs. The primary outcome measure was the proportion of women with bleeding controlled with the trial regimen without recourse to further treatment. Secondary outcomes including the rate of severe PPH, mean/median blood loss, use of additional uterotonics and/or interventions side effects, and acceptability were also recorded.

**Results:**

Of the 258 women who received treatment for PPH, 128 received placebo and misoprostol and 130 received TXA and misoprostol. The proportion of women who had active bleeding controlled with trial drugs alone and no additional interventions was similar in both groups: 77(60.2%) placebo; 74 (56.9%) TXA, *p* = 0.59). Use of other interventions to control bleeding, including uterotonics, did not differ significantly between groups. Median blood loss at PPH diagnosis was 700 ml in both groups. Uterine atony alone or in addition to another cause contributed to over 90% of PPH cases reported (92.2% placebo vs. 91.5% TXA), other causes included perineal and cervical lacerations and retained placenta. Reports of side effects and acceptability were similar in the two groups.

**Conclusion:**

Adjunct use of oral TXA with misoprostol to treat PPH resulted in similar clinical and acceptability outcomes when compared to treatment with misoprostol alone.

**Trial registration:**

This trial was registered with ClinicalTrials.gov, number NCT02805426. Registered on 3 September 2016.

## Plain English summary

Excessive bleeding after childbirth – postpartum hemorrhage (PPH) – is a complication that can occur without warning and can quickly lead to death. Timely treatment strategies are urgently needed wherever women deliver. Tranexamic acid (TXA) is a blood clot stabilizer used routinely for reduction of blood loss in surgery and trauma. It has shown promise in reducing the risk of death from bleeding after childbirth when given by intravenous (IV) administration within 3 h of delivery, and is recommended in clinical guidelines (WHO 2017). The route and time-dependent administration make TXA out of reach for most women who experience primary PPH in settings where IV administration is not feasible and transfer within 3 h is unlikely. TXA is widely available in tablet form at low cost and is stable at room temperature, creating a potential opportunity for its use as part of a PPH management package in lower level health facilities and home births. This trial explored the potential benefit of oral TXA when used as an adjunct to sublingual misoprostol to treat PPH following vaginal delivery. Two hundred and fifty-eight women diagnosed with PPH were randomly assigned to receive sublingual misoprostol and either oral TXA or placebo. Providers measured blood loss for 2 h after administration of the medicines and recorded suspected cause of PPH, blood loss, additional interventions, and side effects. The findings suggest that the addition of oral TXA did not confer any substantial advantage in treating primary PPH when compared to treatment with misoprostol alone.

## Introduction

Obstetric hemorrhage contributes to approximately 25% of maternal deaths worldwide [1]. Despite systematic use of prophylaxis, PPH still occurs in 3–10% [2, 3], of deliveries.

Tranexamic acid, a synthetic derivative of the amino acid lysine, is an anti-fibrinolytic agent that acts by blocking the lysine binding sites on plasminogen [4]. TXA significantly reduces postoperative blood loss and the need for transfusions following surger y[5–8]^.^.A multi-site trial demonstrated that IV TXA (1 g) administered immediately after the onset of postpartum bleeding and within 3 h of birth reduces death among women with PPH [9]. In fact, WHO recommends that IV TXA should be administered to all diagnosed cases of PPH, regardless of cause [10].. While these findings confirm that IV TXA is effective in in helping manage PPH, questions remain as to whether a tablet formulation administered orally is also an effective treatment option. Currently, no information is available about its effectiveness in oral formulation for PPH treatment. Oral TXA has been explored for PPH prophylaxis and may be effective when combined with misoprostol when administered postpartum [11]. If oral TXA were helpful in reducing blood loss after delivery for women experiencing PPH, it could be administered by mid and low level providers at lower level health facilities or home births, where parenteral administration is not feasible and transfer to higher level of care may be delayed. TXA could then serve as a complement to misoprostol, an E1 prostaglandin which is effective in controlling PPH [2, 3] cause by atony. The two drug regimen of oral TXA and misoprostol might improve the efficacy of treatment of PPH in low resource settings, where a large proportion of deaths from PPH occur [1]. This trial aimed to assess the proportion of women with bleeding controlled when oral tranexamic acid is used in conjunction with misoprostol for treatment of PPH.

## Methods

This individually, randomized, double-blind, placebo-controlled trial enrolled participants from October 25, 2016 to January 19, 2018 in four secondary and tertiary level hospitals in Senegal (2) and Vietnam (2). During the course of the trial, due to slower than expected enrollment, one site in Senegal was replaced with a new site in Vietnam in June 1, 2017. To be eligible, women had to deliver vaginally and give written informed consent prior to delivery. Women were excluded if there was a history of thrombosis or a clear contraindication for tranexamic acid such as a known allergy. Per standard of care, women received (either 5 or 10 IU) oxytocin prophylactically. After delivery, blood loss was measured using a plastic drape with a calibrated funnel (MEDIPRO©, Vietnam) that was placed under the woman’s buttocks immediately after delivery of the baby for a minimum of 30 min or until active bleeding ceased. PPH was diagnosed if blood loss reached 700 ml on the drape. Providers could also diagnose PPH based on clinical signs and symptoms (such as heart rate or blood pressure). Women diagnosed with PPH were randomized to receive the next sequential trial drug packet, which consisted of 1950 mg oral tranexamic acid (3 × 650 mg, AMRING Pharmaceuticals) or placebo and 800 mcg (four × 200 mcg) misoprostol (GyMiso®, HRA Pharma, France) administered sublingually. Women were asked to swallow the oral TXA or oral placebo tablets immediately after diagnosis with water before placing the four misoprostol tablets under the tongue.

The oral dose of tranexamic acid selected for this study was based on available pharmacokinetic data in the literature including a previous study reporting on the safety of administering 2000 mg dose [12]. While much higher doses have been shown to be safe and are recommended for heavy bleeding during menses (1300 mg × 3 daily), the selected dose for this study was also based on practical considerations, taking into account the number of pills that would be needed based on the tablet dose available in Vietnam, where study drugs were procured. .

The randomization scheme was computer-generated in blocks of ten and maintained by Gynuity Health Projects. The allocation ratio between placebo and TXA was equal overall but randomly varied within blocks. Trial staff including providers and women were masked to treatment assignment. The trial was unblinded after all trial data were collected. Periodic monitoring ensured that each hospital followed the numerical sequence of the boxes and that masking was successful.

For all PPH cases, blood loss was recorded at five intervals: at treatment, 20 min-, 40 min-, 1 h-, and 2 h- after treatment. If the woman was stable, providers were asked to wait 20 min after administering trial treatment before considering additional interventions for the PPH, although the administration of any additional intervention at any time point was documented. Additional interventions and the cause of PPH (as determined by the provider) were documented. At the time of discharge from the hospital, participants were asked about side effects, acceptability and satisfaction with the trial treatment.

The primary outcome was the proportion of women for whom bleeding was controlled with just the trial regimen (placebo or 1950 mg TXA, followed by 800 mcg misoprostol) without recourse to additional treatment. Controlled bleeding was subject to provider assessment. We hypothesized that bleeding would stop among 89% of women in the placebo group (misoprostol alone), as demonstrated in previous trials on the efficacy of misoprostol to treat PPH [2]. We estimated that an additional 8% of women who received the TXA in addition to the misoprostol would experience cessation of bleeding with no other intervention. Based on these assumptions (89% vs 97%), a sample of 250 PPH cases (125 per group) was required for a one-sided test with 80% power, alpha = 0.05.

Data were collected and recorded by staff trained in trial procedures and reviewed by coordinators at each hospital. Data were entered in SPSS 15 software (IBM, Chicago, IL, USA). All data were entered and analyzed using SPSS 19 software (IBM, Chicago, IL, USA).

The trial was planned and analyzed as intent to treat (ITT). Univariate analysis reported on demographic variables. Bivariate analyses, stratified by trial arm (ITT) and by actual receipt of and compliance with the intervention tested the primary and secondary outcomes.

The protocol was approved by National Council on Health Research, National Ethical Committee, Ministry of Health and Prevention, Senegal and in Vietnam; Ethics Committee in Biomedical Research of Hung Vuong Hospital and Ethics Committee in Biomedical Research of the National Hospital and is reported in accordance with the revised CONSORT statement [13]. An independent Data Safety Monitoring Board (DSMB) reviewed the dataset for safety concerns when two-thirds of the PPH cases had been enrolled.

## Results

**Fig. 1:**
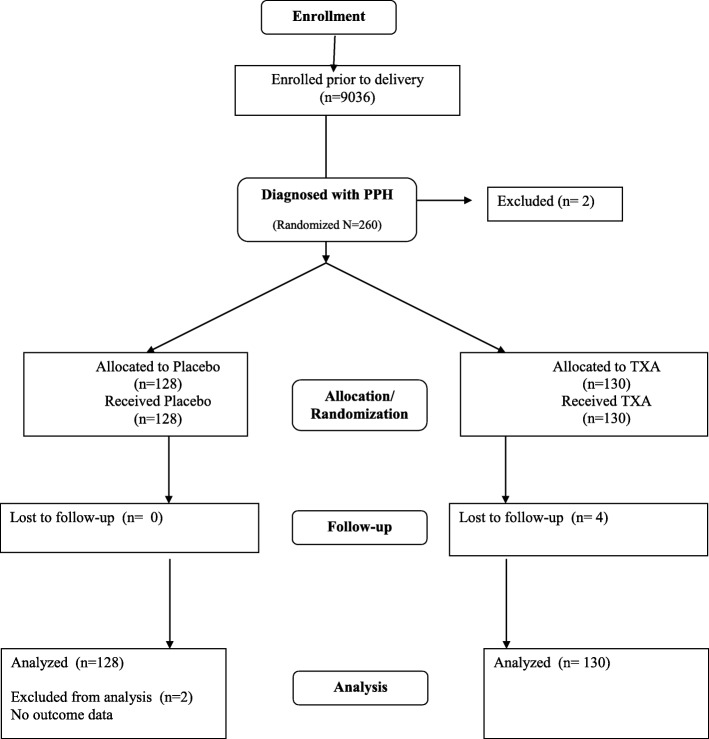
Consort flow chart

Of the 9036 participants, 260 (3%) were diagnosed with PPH. A total of 258 women were randomized and included in the analysis: 128 in the placebo arm and 130 in the TXA group. Two PPH cases were eligible but not randomized: in one case the woman was unconscious and could not take oral medication, and the second woman experienced secondary PPH 9 days after the delivery. All 258 received the 800mcg sublingual misoprostol as part of the study intervention. (Fig. 1). There were no differences in baseline or delivery characteristics between the two groups (Table 1). Among women who received placebo, active bleeding was controlled without recourse to additional interventions in 77 (60.2%) of cases compared to 74 (56.9%) in the TXA group (*p* = 0.59) (Table 2) RR = 0.936, 95% CI 0.734–1.194) Of the women who received interventions in addition to the trial regimen, approximately one-third received additional uterotonics (placebo: 39 (30.5%); TXA treatment: 45 (34.6%) *p* = 0.51) (Table 2). Receipt of additional IV TXA (placebo: 16 (12.5%); TXA: 17 (13.1%)) was similar across groups. In most instances the additional interventions were administered within 20 min after administration of trial regimen (placebo: 39 (78%); TXA: 43 (76.8%)) (data not shown). Disaggregated results show no significant differences in outcomes associated with cause of PPH or uterotonics received prior to third stage of labor (data not shown).

**Table 1:** Baseline and delivery characteristics among vaginal births (PPH cases only)

	Miso + Placebo *N* = 128	Miso + TXA *N* = 130
Age *Median (range)*	28 (19–48)	28 (17–40)
Parity	0.61	0.85
Woman experience previous PPH	3 (2.3)	7 (5.4)
Singleton birth	125 (97.7)	125 (96.2)
Neonatal deaths	1 (0.8)	0 (0.0)
Episiotomy	117 (91.4)	113 (86.9)
Uterotonic to induce labor	11 (8.6)	13 (10.0)
Uterotonic to augment labor	39 (30.5)	39 (30.0)
Oxytocin prophylaxis	128 (100)	130 (100)
*IM*	123 (96.1)	118 (90.8)
Manual removal of placenta^a^	5 (3.9)	11 (8.5)

There were no statistically significant differences between the two groups for any of these variables

^a^ did not involve transfer to theater. No other complications were noted for these women

**Table 2:** Primary Outcome, treatment outcomes and interventions

	Placebo *N* = 128	TXA *N* = 130
Bleeding controlled with treatment only –(no additional intervention)^a^	77 (60.2)	74 (56.9)
Bleeding controlled with treatment only –(no additional serious intervention)^b^	102 (79.7)	108 (83.1)
Additional interventions		
Oxytocin	37 (28.9)	39 (30.0)
*IV*	25 (19.5)	25 (19.2)
*IM*	12 (9.4)	14 (10.8)
Ergometrine	34 (26.6)	34 (26.2)
Syntocinon	1 (0.8)	1 (0.8)
Carbetocin *IV*	10 (7.8)	14 (10.8)
Misoprostol	0 (0.)	1 (0.8)
TXA IV	16 (12.5)	17 (13.1)
Uterine evacuation (MVA)	0 (0.0)	1 (0.8)
Bimanual compression	8 (6.3)	8 (6.2)
Suturing	111 (86.7)	108 (83.1)
Uterine packing	15 (11.7)	10 (7.7)
Blood transfusion	13 (10.2)	12 (9.2)
Uterine artery ligature	1 (0.8)	0 (0)
Hysterectomy	1 (0.8)	0 (0)
Tissue repair	2 (1.6)	1 (0.8)
Plasma expanders	4 (3.1)	3 (2.3)

There were no statistically significant differences between the two groups for any of these variables

^a^additional interventions include: uterotonics, TXA, bimanual compression, uterine evacuation, uterine packing, blood transfusion, uterine artery ligation, hysterectomy, tissue repair, plasma expanders. Suturing and administration of IV fluids were excluded

^b^ additional serious interventions calculated as all interventions excluding uterotonics, TXA, bimanual compression, suturing and fluids (serious interventions only)

Median blood loss at diagnosis for women who received study treatment was 700 ml (range 500-1500 ml) (Table 3). Only 6 women (2.3%) were diagnosed with severe PPH (blood loss ≥1000 ml).

**Table 3:** PPH diagnosis and blood loss

	Miso + Placebo *N* = 128	Miso + TXA *N* = 130
Median Blood loss at PPH diagnosis	700 (500–1200)	700 (500–1500)
Reason for PPH		
*Uterine atony (among causes)*	118 (92.2)	119 (91.5)
*Uterine atony alone*	91 (71.1)	89 (69.5)
*Uterine atony + other cause*^*a*^	27 (21.3)	30 (23.4)
*Non atonic cause*	9 (7.1)	9 (7.0)
Median blood loss at treatment	700 (500–2000)	700 (500–1500)
Median blood loss at 20 min post treatment	750 (500–2200)	750 (550–1600)
Median blood loss at 40 min post treatment	800 (500–2300)	800 (550–2000)
Median blood loss at 1 h post treatment	800 (500–2300)	800 (550–2000)
Median blood loss at 2 h post treatment	800 (500–2300)	800 (550–2000)
Time to bleeding controlled post treatment		
Mean	33 min (0-2 h)	33 min (0-2 h)
Median	20 min	20 min
*Mean time to bleeding controlled post*	*23 min (N = 74)*	*28 min (N = 74)*
*treatment- TXT DRUG ONLY*	*Median: 20 min*	*Median: 20 min*
*Mean time to bleeding controlled post*	*48 min (N = 52)*	*41 (N = 54)*
*treatment- ADDITIONAL INTERVENTIONS*	*Median: 30 min*	*Median:30 min*

There were no statistically significant differences between the two groups for any of these variables

^a^Other causes included cervical or perineal lacerations and retained placenta

Uterotonics had been used to induce labor in approximately 9% of the cases and augment in 30% of the cases. Over 90% of women had episiotomy, and all women were given oxytocin prophylaxis during the third stage of labor. Characteristics of the enrolled PPH cases were comparable in the two study groups. Uterine atony either alone or in addition to other causes cited contributed to the majority of PPH cases (placebo: 118 (92.2%); TXA: 119 (91.5%)). Approximately 70% of women experienced uterine atony as the sole cause of PPH (placebo: 91 (71.7%); TXA: 89 (69.5%)) while 21.3% (*n* = 27) and 23.4% (*n* = 30) for placebo and TXA respectively, had PPH due to uterine atony in addition to perineal lacerations, cervical lacerations or retained placenta. 7.1% (*n* = 9) and 7.0% (*n* = 9) in the placebo and TXA groups respectively, had a reported PPH due to non-atonic causes (Table 1). Median time to PPH treatment after birth was 10 min (same in each arm) with only 6.3% (placebo) and 7.7% (TXA) administered treatment more than 1 h after childbirth (data not shown).

The side effect profile was similar in the two trial groups. Shivering was reported as the most frequent side effect experienced [placebo: 81 (63.8%); TXA: 82 (63.6%)] followed by fever [placebo: 40 (31.3%); TXA: 33 (25.4%)] (Table 4). In both groups, women reported that treatment was acceptable (Table 4). One participant in the placebo arm who experienced atonic PPH underwent a hysterectomy 4 days post-delivery. In this case, oxytocin was used to induce and augment the delivery. Approximately an hour after PPH diagnoses, the placenta was removed manually and the uterus packed. The woman received IM oxytocin, ergometrine, IV TXA and 2 units of blood. Shortly after receipt of these interventions a uterine artery ligation and B-lynch were performed. After her 2-day hospital stay, the woman was transferred to a cardiac unit because of cardiac concerns where the hysterectomy was performed 2 days later after diagnosis of uterine necrosis. She was discharged in stable condition. There were no deaths reported among trial participants.

**Table 4:** Side effects

	Miso + Placebo *N* = 128	Miso + TXA *N* = 130
Side Effects experienced:		
Shivering	81 (63.8)	82 (63.6)
Fever	40 (31.3)	33 (25.4)
Nausea	13 (10.2)	9 (7.0)
Vomiting	7 (5.5)	6 (4.7)
Diarrhea	1 (0.8)	1 (0.8)
Fainting	1 (0.8)	2 (1.6)
Acceptability (reported by women)	*N* = 119	*N* = 121
*Very acceptable or acceptable*	107 (89.9)	113 (93.3)
*Neutral*	7 (5.7)	4 (3.3)
*Very unacceptable or unacceptable*	5 (4.2)	4 (3.3)

There were no statistically significant differences between the two groups for any of these variables

## Discussion

This trial was designed to explore whether the addition of oral TXA to misoprostol treatment of PPH would produce improved outcomes. The rate of additional interventions employed to curb bleeding and mean blood loss post-treatment was not significantly different among women who received oral TXA in addition to misoprostol compared to women who did not receive TXA. These findings suggest that the addition of oral TXA did not confer any substantial advantage in treating PPH. It may be possible that TXA offers limited additional clinical benefit related to serious outcomes.

Previous trials have documented that 9 out of 10 women experience cessation of bleeding within 20 min after administration of misoprostol without recourse to additional interventions [3]. Although median time to bleeding cessation for both groups was also 20 min in our study, almost 80% of women who received additional interventions received them less than 20 min after study treatment, possibly limiting the observed effect of trial drugs alone. Additional analysis, excluding women who received treatment prior to 20 min, continues to show no significant differences between treatment groups [placebo 78/89 (87.6%); TXA 74/87 (85.1%)].

Whereas IV TXA is recommended for use in the treatment of PPH, oral TXA may not confer the same effect as IV administration, which allows for much greater bioavailability [11]. Therefore, it is possible that the misoprostol treatment (in addition to other interventions), may have effectively controlled blood loss before oral TXA could take effect. Moreover, studies have highlighted the importance of early use to treat PPH [14].Although in the the large multisite WOMAN trial, IV TXA was shown to reduce death due to blood loss [9], there was no benefit if TXA was administered more than 3 h postpartum. Given the later onset of action, oral TXA may be most effective when administered earlier in delivery.

The oral TXA dose administered in this study was based on the established safety a 2000 mg dose [12]. Given current recommendations for higher doses (used for management of heavy menstrual bleeding) the lower study dose used may have constituted a limitation in examining the true potential of oral TXA. Nevertheless, a study testing 1000 mg oral TXA at the end of the first stage of labor followed by misoprostol showed that this regimen was associated with lower blood loss when compared to prophylactic IV oxytocin [11].

Another limitation of our study is that the rates of intervention were very different from our assumptions for sample size calculation (above). We estimated that bleeding would be controlled with no additional intervention in 89% of cases in the placebo group, when in fact this was the case for only 60%. We aimed to conduct an exploratory study of a pill-based regimen in a location where other treatments were readily available in case they were needed. The unintended consequence of the high use of additional interventions at these sites may have made it difficult to assess the true effect of the study medicines. As in the WOMAN trial, where the authors argue that interventions carried out prior to TXA treatment may have diluted its effect, it is possible that outcomes might be different with a larger sample and in other delivery settings with more limited options for PPH management.

Consistent with the literature [15], atony contributed to over 90% of the PPH cases and was the primary cause of PPH for approximately 70% of women in this cohort. These findings reconfirm the importance of ensuring good access to high quality uterotonics for management of hemorrhage. The management practices documented in this study, however, also reveal the potential for over-use of PPH medicines that are becoming increasingly available. For example, 80.4% (*n* = 86) of the women who received additional interventions received additional uterotonics, and 76.7% (*n* = 66) of these women received 2 or more uterotonic drugs for PPH treatment. This tally does not include the uterotonics also given for induction/augmentation and prophylaxis or the misoprostol treatment all women received. Administration of multiple doses and potential overuse of available medicines deserve greater attention and evaluation in relation to women’s quality of care, clinical outcomes (additional treatments with uterotonics did not appear to have an overall effect on blood loss), and resources and costs to health systems.

## Conclusion

While trial findings suggest that oral TXA may not have a significant effect in addressing PPH, options that treat non-atonic causes deserve consideration. International guidelines, clearly recommend that IV TXA be administered to women diagnosed with PPH and should be made available as part of a standard treatment package. It nevertheless remains imperative to explore simple options to manage all causes of PPH and to manage PPH related morbidity, especially when access to IV therapy or surgical interventions is limited.

## Data Availability

The datasets used and/or analyzed during the current study are available from the corresponding author on reasonable request.

## Abbreviations

PPH: Postpartum hemorrhage
TXA: Tranexamic acid
